# Influence of Artery Straightening on Local Hemodynamics in Left Anterior Descending (LAD) Artery after Stent Implantation

**DOI:** 10.1155/2020/6970817

**Published:** 2020-05-22

**Authors:** Pengfei Liu, Xiaoyan Deng, Xiao Liu, Anqiang Sun, Hongyan Kang

**Affiliations:** Key Laboratory for Biomechanics and Mechanobiology of the Ministry of Education, School of Biological Science and Medical Engineering, Beijing Advanced Innovation Center for Biomedical Engineering, Beihang University, Beijing 100083, China

## Abstract

**Objectives:**

The study investigates local hemodynamic environment changes caused by straightening phenomenon and the relationship between straightening phenomenon and in-stent restenosis.

**Background:**

Intravascular intervention is an effective treatment in restoring the normal flow conditions and vascular lumen. Unfortunately, in-stent restenosis often occurs in a subset of patients after stent implantation and limits the success of stent implantation outcomes. The implanted stent usually causes artery straightening locally, rather than coinciding and adjusting to the physiological curve exactly. Artery straightening would apparently modify the artery geometry and therefore alter the local hemodynamic environment, which may result in intimal hyperplasia and restenosis after stenting implantation.

**Methods:**

In the current investigation, we verify the hypothesis that the artery straightening influences the local hemodynamic state using the different 3D CT models. Flow analysis for blood in the left anterior descending coronary artery and the straightening model is simulated numerically.

**Result:**

The current results reveal that the straightening phenomenon alters the distribution of wall shear stress and flow patterns, decreases the wall shear stress (WSS), and increases the oscillatory shear index (OSI) and the relative residence time (RRT), especially at the proximal and distal areas of stenting.

**Conclusions:**

The local straightened geometry established after stent implantation was likely to generate portions of the stenting area to a high risk of neointimal hyperplasia and subsequent restenosis.

## 1. Introduction

Stents are usually implanted into stenotic coronary arteries to improve or restore blood flow environment. However, restenosis has some persistent problems that limit the development of percutaneous coronary intervention [1, 2, 3]. Studies reported that restenosis occurs in 12 % of patients after stent implantation [3].

The mechanisms responsible for restenosis were not yet fully elucidated. Stent geometry and its subsequent effects on localized hemodynamics may cause restenosis [4–6]. Williams et al. reported that reduced vessel compliance and altered distributions of the wall shear stress (WSS) within the stented region could induce restenosis occurrence [7–9]. Besides, previous studies demonstrated that smooth muscle and endothelial cell damage would be implicated as potential factors for stimulating neointimal hyperplasia [8, 10, 11]. There was a putative link between the hemodynamic environment changes and restenosis [12, 13]. Moreover, Regar et al. found that the procedure-specific factors such as implantation technique also can influence restenosis [14].

In addition to these reasons, the existing researches suggested that the straightening phenomenon caused by stent implantation induced changes in local hemodynamic environment and the rate of restenosis occurrence rate [15–18]. However, these studies were just conducted with simple two-dimensional models or three-dimensional idealized models, which cannot illustrate the realistic blood flow environment [7, 19, 20].

The straightening phenomenon is usually due to the different curvatures between stent and lesion vessels, resulting in two evident angle changes near both ends of the stented region. The present study hypothesized that the straightening phenomenon can change the local hemodynamic state and vascular geometry. These changes can increase the possibility of restenosis occurrence and affect the stent surgeries.

In order to verify this hypothesis, the study reconstructed the left anterior descending coronary artery (LAD) and the straightening model based on the realistic computed tomography (CT) images and stent. The normal blow flow condition is used to simulate the local hemodynamic state. This study revealed the changes in local hemodynamics caused by the straightening phenomenon and discussed the relationship between artery straightening and restenosis. The implications of this study help the determination of potential causes of restenosis after stent implantation and the optimization design of the stent.

## 2. Materials and Methods

### 2.1. Reconstruction of Normal and the Straightening Models

**Figure 1:**
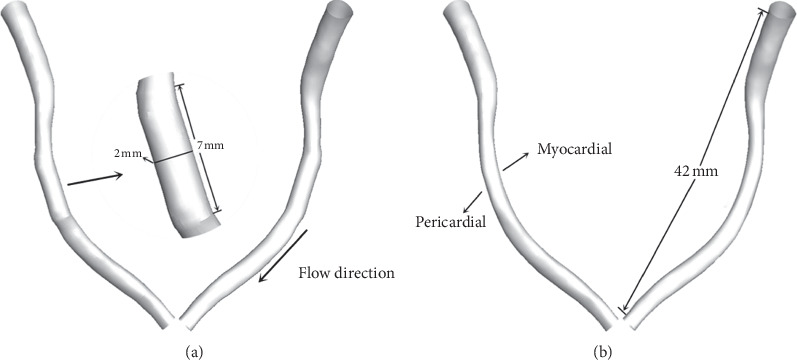
(a) Straightening and (b) normal models. The enlarged image shows the straightening phenomenon caused by stent implantation.

The study reconstructed the realistic coronary artery model based on the CT scan images with Mimics (v15.0, Materialise, Ann Arbor, MI, USA). The CT relevant parameters were described as follows: 0.9 mm slice thickness, 0.45 mm slice increment, 0.324 mm pixel size, a 512 × 512 image resolution, and total 293 [21]. The LAD model was obtained from the coronary artery model by using intercept function. The features of stent structure would be neglected especially after the endothelialization [10, 20]. Therefore, only the artery curve information was considered in the modeling process. The straightening model was obtained from LAD fitting with the stent model. Subsequently, simple smoothing and surfacing processes were applied to the models with Geomagic Studio 2012 (3D Systems, Morrisville, NC, USA). The diameter of LAD is 1.7 mm. Using a stent-to-artery diameter ratio of 1.2, the diameter of the stent was defined as 2 mm, and the length of the stent was 7 mm. The two models are shown in Figure 1.

### 2.2. Meshing

**Figure 2:**
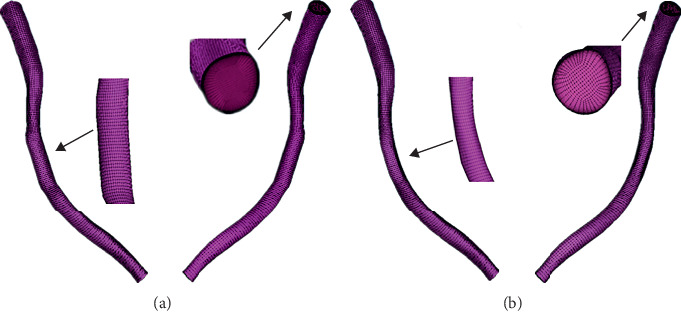
Mesh of the (a) straightening and (b) normal models.

ANSYS ICEM 16.0 (ANSYS, Inc., Canonsburg, PA, USA) software was used to complete the models meshing. Hexahedral elements were used, and the number of boundary layer was set to 10, with the height ratio 1.1 and initial height 0.009 mm. The total node number of the normal model was 116081. The total node number of the straightening model was 112476. Two models' meshes are shown in Figure 2.

### 2.3. Simulation and Boundary Conditions

**Figure 3:**
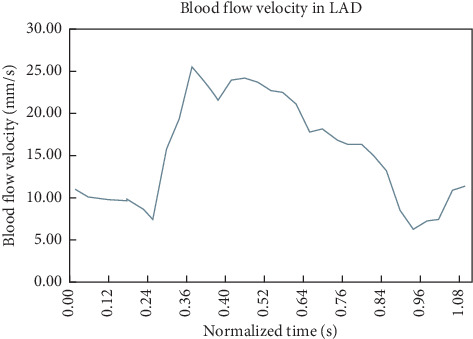
Inlet velocity pulsating flow. A representative waveform describing the average blood flow velocity measured in the proximal portion of a human left anterior descending coronary artery during a cardiac cycle. This waveform was used to complete the time-dependent simulations in the present study.

Simulation calculation was conducted by ANSYS Fluent 16.0 software (ANSYS, Inc*.,* Canonsburg, PA, USA) under the pulsatile flow conditions. Blood was modeled as a Newtonian fluid and assumed to be homogeneous and incompressible [22, 23]. The convergence criterion was set to 0.00001. The vascular wall was postulated to be nonslip of the rigid wall. The inlet condition used the realistic blood flow velocity (Figure 3) [24]. The numerical simulation was conducted based on a three-dimensional incompressible Navier–Stokes equation and the conservation of mass:

(1) $$\begin{array}{c} \rho \left[\frac{\partial u}{\partial t}+\left(u\cdot \nabla \right)u\right]+\nabla p-\mu \nabla^{2}u=0, \end{array}$$

(2) $$\begin{array}{c} \nabla \cdot u=0, \end{array}$$

where **u** and *p* represent the fluid velocity vector and pressure, respectively. *ρ* and *µ* are the density and viscosity of blood (*μ* = 3.5 × 10^−3^ kg/m·s and *ρ* = 1050 kg/m^3^) [25]. SIMPLE algorithm was used to calculate the blood flow velocity, and pressure-based solver was used for pressure correction and to solve the momentum equation. Computation period was set to 1.08 s, each step for 0.008 s, with 136 steps in each cycle. Every cycle was required to obtain a convergence for the transient analysis. The largest number of iterations in every step was 500, and the total steps were 1000. The whole computational process spanned twelve working days.

### 2.4. Hemodynamic Parameters

Postprocessing was conducted with Matlab (The Math Works, Natick, Mass) and Tecplot 360 2013R1 software using the data exported from ANSYS FLUENT 16.0. Three WSS-based hemodynamic parameters (TAWSS, OSI, and RRT) were calculated based on the research by Claudio Chiastra [13].

Wall shear stress (WSS) was defined to be the product of fluid viscosity and shearing velocity of the neighboring vascular wall. We used 1050 kg/m^−3^ as the viscosity of blood. WSS had close connection with blood characters, blood flow velocity, and vascular morphology.

The time-averaged wall shear stress (TAWSS) was the average of WSS in the entire cardiac cycle of WSS. TAWSS was described as the characteristics of WSS in pulsatile flow. TAWSS was calculated as follows:

(3) $$\begin{array}{c} \text{TAWSS}=\frac{1}{T}\int_{0}^{T}\left|\mathrm{WSS}\left(s,t\right)\right|\cdot dt, \end{array}$$

where T is the duration of the cardiac cycle and **s** is the position on the vascular wall.

The oscillatory shear stress index (OSI) was the nondimensional parameter; it indicated the magnitude of WSS fluctuations during a cardiac cycle. It is defined as follows:

(4) $$\begin{array}{c} \text{OSI}=0.5\left[1-\left(\frac{\left|\left(1/T\right)\int_{0}^{T}\text{WSS}\left(s,t\right)\cdot \text{d}t\right|}{\left(1/T\right)\int_{0}^{T}\left|\text{WSS}\left(s,t\right)\right|\cdot \text{d}t}\right)\right]. \end{array}$$

The high OSI leads to the lack of endometrial cells function and the decisive factor to change cellular structure cyclic stress.

The relative residence time (RRT) is introduced:

(5) $$\begin{array}{c} \text{RRT}=\frac{1}{\left(1-2\cdot \text{OSI}\right)\cdot \text{TAWSS}}. \end{array}$$

The RRT is inversely proportional to the magnitude of the TAWSS vector and has obvious connections to the biological mechanisms of atherosclerosis. The OSI modifies the TAWSS effects on the RRT at a given region of the endothelium. Therefore, the RRT parameter includes the effects of both OSI and TAWSS.

## 3. Results

Two reconstruction models were used for simulation calculation; the best convergence cycle of seven cycles was selected to analyze the experiment data. On the basis of the hemodynamic parameters (blood flow velocity, WSS, TAWSS, OSI, and RRT), the study aimed at investigating the influence of straightening phenomenon after stent implantation on local hemodynamic environment in LAD.

### 3.1. The Blood Flow Velocity

**Figure 4:**
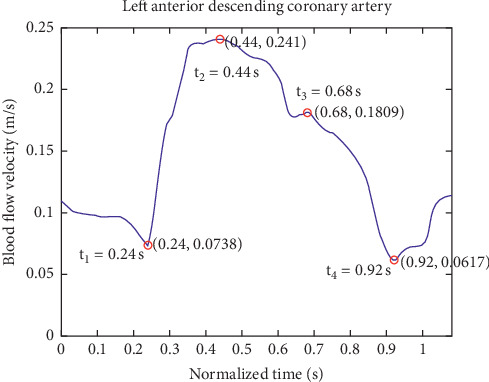
Four typical points during one cardiac cycle. *t*_1_, *t*_2_, *t*_3_, and *t*_4_ are selected from one cardiac cycle and, respectively, represent early systole, peak systole, second peak systole, and end systole.

Four points from one cardiac cycle are shown in Figure 4; early systole (*t*_1_), peak systole (*t*_2_), second peak systole (*t*_3_), and end systole (*t*_4_) were selected from one cardiac cycle.

**Figure 5:**
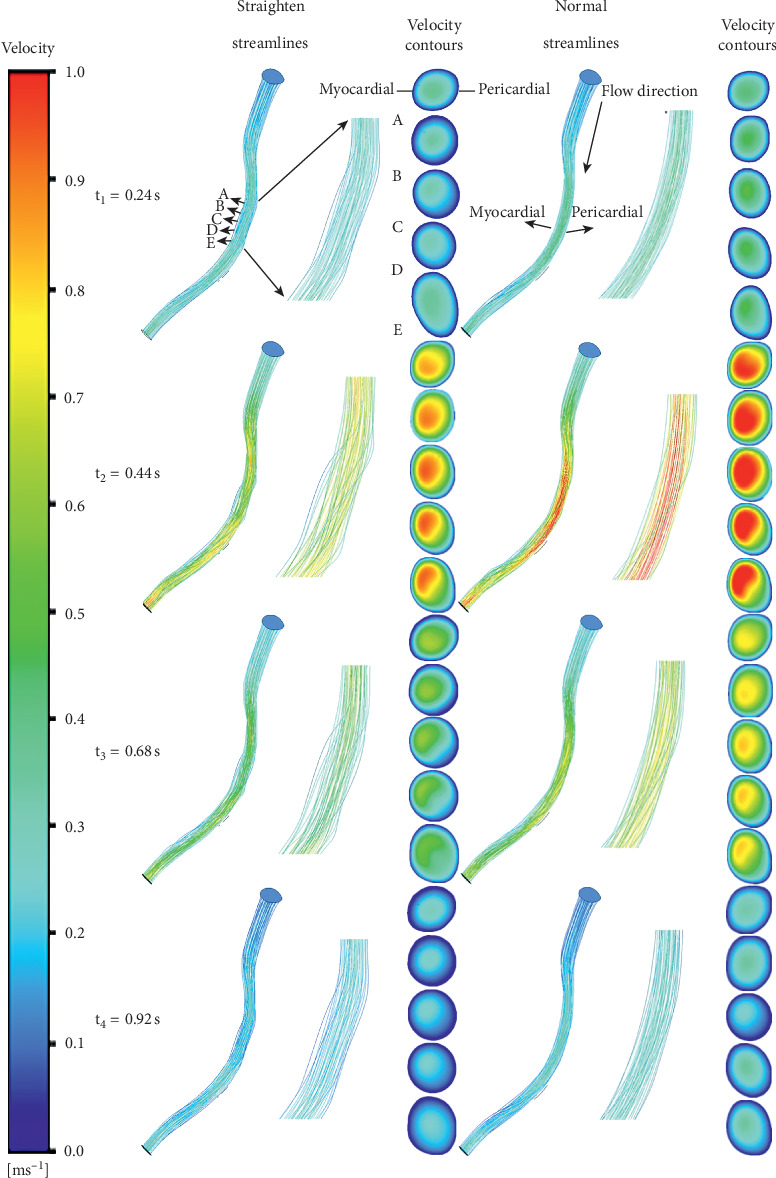
The streamlines near the left anterior descending coronary artery at four typical points with the various models. Cross sections are velocity contours in different moments of the different models. A, B, C, D, and E are selected from the models, and the first point is located at the inlet of the stented region. The distance between two points is 1 mm, and these points are also shown. Slicing in these points along the direction perpendicular to the centerline forms the cross sections.

Figure 5 shows the velocity streamlines in normal and straightening models. There was no obvious difference in the flow patterns and velocity between the models except the stenting area. In the stented area, the blood velocity of the straightening model obviously decreased. The highest blood flow velocity and the maximum difference of velocity between models emerged at the *t*_2_ moment. In contrast, the *t*_4_ moment had the lowest blood flow velocity and the minimum difference of velocity between models. The flow pattern of the straightening model obviously altered at time *t*_1_ and time *t*_3_. According to the velocity contours, the velocity near the pericardial surface was higher. The difference of velocity distribution between two models increased from *t*_1_ to *t*_3_, and the maximum disparity occurred at time *t*_3_.

### 3.2. WSS and TAWSS

**Figure 6:**
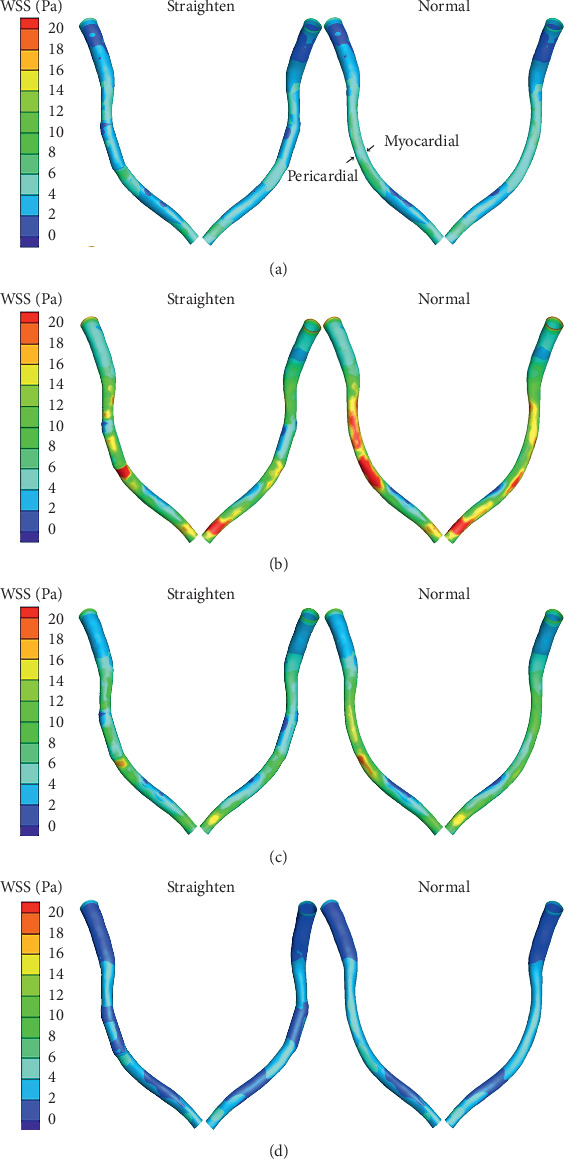
Counters of WSS at four typical points: (a) *t*_1_ = 0.24 s; (b) *t*_2_ = 0.44 s; (c) *t*_3_ = 0.68 s; (d) *t*_4_ = 0.92 s.

Contours of the WSS at the four points are shown in Figure 6. The distributions of WSS between models except the stented area were similar. In the stented area, the distributions of WSS were significantly altered, and the WSS was reduced by the straightening phenomenon. The WSS of the straightening model illustrates a gradient increase along the pericardial surface. To compare with other points, the maximum change in WSS between two models occurred at the *t*_2_ moment, and the minimum difference occurred at time *t*_4_. From the straightening model, the highest average WSS was 8.77 Pa at time *t*_2_, and the lowest average WSS was 2.20 Pa at time *t*_4_. Meanwhile, from the normal model, it increased to 11.27 Pa at time *t*_2_ and 2.68 Pa at time *t*_4_.

**Figure 7:**
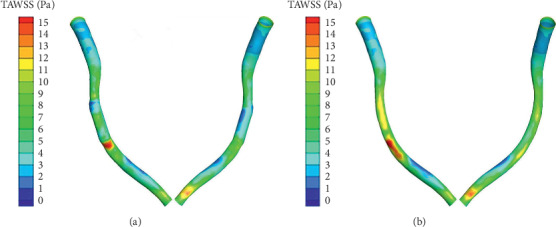
Counters of TAWSS: (a) straightening; (b) normal.

The distribution of TAWSS is described in Figure 7 (TAWSS counters on different models), and similar distribution of TAWSS was observed compared with above-described WSS distribution. Within the stented area, the TAWSS of the straightening model significantly decreased, especially at the inlet and outlet of the stenting region and the pericardial surface. Examination of TAWSS as a function of normalized axial length revealed that TAWSS of the straightening model was greater at the pericardial compared to the myocardial luminal surface. The largest TAWSS difference occurred at the inlet area.

### 3.3. OSI and RRT

**Figure 8:**
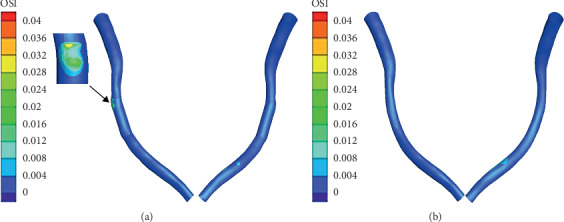
Counters of OSI: (a) straightening; (b) normal.

Figure 8 shows the contours of OSI on the different models. Generally, the areas of high OSI were observed in areas of low WSS where the direction changed frequently. The significantly higher OSI of the straightening model was observed at the inlet of the stented area compared to the normal (shown in enlarge image).

**Figure 9:**
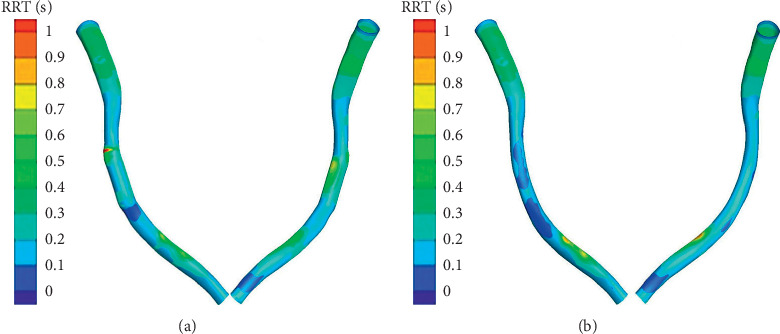
Counters of relative residence time (RRT): (a) straightening; (b) normal.

The contours of RRT under different conditions are shown in Figure 9. The straightening phenomenon increased RRT values within the stented area, especially the RRT at the inlet of the stent and the pericardial surface. From the straightening model, the difference of RRT on both sides was higher. The highest RRT of the straightening model was calculated at the inlet of the stent along the myocardial surface, and the maximum RRT difference between two models occurred in this region. Beyond all that, the straightening phenomenon had a serious influence on the distribution of RRT.

## 4. Discussion

Stenting is used as an important treatment for critical artery stenosis. However, postoperation complications are still perplexing the patients over time, for example, neointimal hyperplasia and subsequent restenosis [7, 8, 9, 17, 26]. The reasons causing the restenosis are still being explored. Numerous studies have been conducted to investigate the potential influences including stent types, carrying drugs, and many postoperative and intraoperative factors [26–28]. As the compliance of the stent usually does not adjust to the curved artery, artery “straightening” is easily formed at the stented areas, potentially changing the local blood flow features and consequently influencing arterial cells behaviors and inducing tissue remodeling. It would be very useful and necessary to investigate the detailed hemodynamic changes in the straightened areas after stenting. Some studies focused on the straightening of vascular caused by stent implantation, but the relationship between the straightening phenomenon and restenosis was unclear [15–17, 28]. In these published studies, idealized cylindrical models were usually used to examine flow patterns through stented vessels with “straightening” [7, 19, 20]. However, the detailed local hemodynamic features changed by straightening phenomenon in realistic 3D models was not clear.

In the last 5 decades, the low wall shear stress hypothesis of atherosclerosis proposed by Caro et al. has been validated [29]. The connection between low WSS and high intimal proliferation had been further certified in rodents [30]. The vascular regions subjected to WSS below 0.5 Pa have been shown to strongly correlate with sites of intimal thickening [31, 32]. Low TAWSS was thought to be associated with regions of cellular proliferation and the potential factors for the development of neointimal.[33]. The high OSI created greater endothelial cells proliferation, and increased RRT had obvious connections with biological mechanisms of atherosclerosis [18]. Moreover, Colombo pointed that abnormal blood flow patterns can promote inflammation, endothelial cells proliferation, and thrombosis [34].

In study, the straightening phenomenon caused by stent implantation was an potential factor to the restenosis. Normal and straightening models based on CT images were reconstructed, and blood flow parameters in LAD were used as boundary conditions. The numerical simulation results revealed the straightening phenomenon altered the lumen geometry and consequently changed blood flow patterns in the local areas. The velocity and WSS in the stented area decreased after the straightening phenomenon. Besides, the RRT and OSI increased on the straightening model. The significant changes in parameters happened especially at the proximal and distal end of stented areas and the pericardial surface. As the low WSSs, high RRTs, and high OSIs were all indexes of restenosis subsequently [18], the straightening phenomenon hereby would induce a hemodynamic environment in LAD that promotes these conditions. Therefore, we conclude that the straightening phenomenon could affect the hemodynamic environment in the LAD and expose the endothelial cells to low WSS, high OSI, and high RRT conditions. These can make stented area more likely to cause the restenosis and neointimal hyperplasia, especially at the proximal and distal ends of the stented area and the pericardial surface. This conclusion coincided with the findings of Wentzel and colleagues who observed clinical evidence of restenosis in [16].

Because some assumptions and simplification have been used in this study (e.g., the rigid wall assumption and neglect of detailed stent strut features), the results may not perfectly describe the hemodynamic environment in the straightened areas. However, the present study can still provide some clues to investigate the physiological and pathological impacts of straightening phenomenon after stent implantation.

In the future, at least two issues should be highly concerned. The first one is to analyze the artery wall stress and strain in the straightened areas. Because the arterial wall remodeling could be significantly regulated by the stress and strain in the artery [35]. The second one is the drug delivery and deposition characteristics in the special hemodynamic environment caused by straightening phenomenon. Drug eluting stent (DES) is now being prevalently used in clinics, and previous studies had proved the high correlation between local blood flow patterns and drug delivery [12, 36, 37]. Thus, investigating the drug delivery and deposition characteristics in straightened areas will be important in optimizing DES stenting.

## 5. Conclusion

This study investigated the local hemodynamic environment changes caused by straightening phenomenon. And we explored the relationship between the straightening phenomenon and the restenosis. The straightening phenomenon would lead to the decrease in WSS, TAWSS, and blood flow velocity and increase in RRT and OSI. This study concluded that the regional geometry of straightening phenomenon established after stent implantation is likely to lead to portions of the stenting area to a high risk of neointimal hyperplasia and subsequent restenosis (especially in the inlet and outlet of the stent and the pericardial surface). This study could help elucidate the mechanism of restenosis and promote the development of stent technology. This study can also serve as a reminder of the potential risks and provide useful guides in the stent deployment procedures.

## Data Availability

The data used to support the findings of this study are included within the article.

## Abbreviations

CT: Computed tomography
LAD: Left anterior descending
WSS: Wall shear stress
TAWSS: Time-averaged wall shear stress
OSI: Oscillatory shear stress index
RRT: Relative residence time.
